# An *in vitro* α-neurotoxin—nAChR binding assay correlates with lethality and *in vivo* neutralization of a large number of elapid neurotoxic snake venoms from four continents

**DOI:** 10.1371/journal.pntd.0008581

**Published:** 2020-08-28

**Authors:** Kritsada Pruksaphon, Kae Yi Tan, Choo Hock Tan, Pavinee Simsiriwong, José María Gutiérrez, Kavi Ratanabanangkoon

**Affiliations:** 1 Department of Microbiology, Faculty of Medicine, Chiang Mai University, Chiang Mai, Thailand; 2 Department of Molecular Medicine, Faculty of Medicine, University of Malaya, Kuala Lumpur, Malaysia; 3 Department of Pharmacology, Faculty of Medicine, University of Malaya, Kuala Lumpur, Malaysia; 4 Laboratory of Immunology, Chulabhorn Research Institute, Bangkok, Thailand; 5 Instituto Clodomiro Picado, Facultad de Microbiología, Universidad de Costa Rica, San José, Costa Rica; 6 Department of Microbiology, Faculty of Science, Mahidol University, Bangkok, Thailand; Liverpool School of Tropical Medicine, UNITED KINGDOM

## Abstract

The aim of this study was to develop an *in vitro* assay for use in place of *in vivo* assays of snake venom lethality and antivenom neutralizing potency. A novel *in vitro* assay has been developed based on the binding of post-synaptically acting α-neurotoxins to nicotinic acetylcholine receptor (nAChR), and the ability of antivenoms to prevent this binding. The assay gave high correlation in previous studies with the *in vivo* murine lethality tests (Median Lethal Dose, LD_50_), and the neutralization of lethality assays (Median Effective Dose, ED_50_) by antisera against *Naja kaouthia*, *Naja naja* and *Bungarus candidus* venoms. Here we show that, for the neurotoxic venoms of 20 elapid snake species from eight genera and four continents, the *in vitro* median inhibitory concentrations (IC_50s_) for α-neurotoxin binding to purified nAChR correlated well with the *in vivo* LD_50s_ of the venoms (R^2^ = 0.8526, *p* < 0.001). Furthermore, using this assay, the *in vitro* ED_50s_ of a horse pan-specific antiserum against these venoms correlated significantly with the corresponding *in vivo* murine ED_50_s, with R^2^ = 0.6896 (*p* < 0.01). In the case of four elapid venoms devoid or having a very low concentration of α-neurotoxins, no inhibition of nAChR binding was observed. Within the philosophy of 3Rs (Replacement, Reduction and Refinement) in animal testing, the *in vitro* α-neurotoxin-nAChR binding assay can effectively substitute the mouse lethality test for toxicity and antivenom potency evaluation for neurotoxic venoms in which α-neurotoxins predominate. This will greatly reduce the number of mice used in toxicological research and antivenom production laboratories. The simpler, faster, cheaper and less variable *in vitro* assay should also expedite the development of pan-specific antivenoms against various medically important snakes in many parts of the world.

## Author summary

Snakebite envenomation is an important public health problem recognized by the World Health Organization (WHO) as a neglected tropical disease affecting about 2 million of poor people of the tropical world. The most effective therapy is the timely administration of efficacious antivenoms which are usually produced in horses. The serum/plasma of horse immunized with snake venoms is purified and tested for its efficacies in neutralizing the target venoms. The neutralization is assayed using mice injected with the venom together with the antivenom. This assay requires about 60 mice for each pair of venom and antivenom. The assay is expensive, laborious, giving highly variable results and is objected on ethical and religious grounds. The present study involves the development of an *in vitro* assay involving the binding of a snake neurotoxin to a soluble receptor protein called nicotinic acetylcholine receptor. It is shown here that this receptor binding assay gave good correlation with the assay using mice. The test tube assay is simpler, faster, cheaper and less variable when compared with the mouse assay and thus could reduce or even replace the use of life animal. Furthermore, it could expedite the development of effective antivenoms against various venomous snakes in many parts of the world.

## Introduction

Snakebite envenomation is an important public health problem recognized by the World Health Organization (WHO) as a neglected tropical disease [1]. It has been estimated that 2 million people in the tropical world suffer these envenomations, resulting in about 20,000–94,000 fatalities annually [2]. The only effective treatment is the timely administration of antivenom. However, currently, effective antivenoms are not widely available and/or affordable in many parts of the world, especially in impoverished rural settings of sub-Saharan Africa and parts of Asia and Latin America [3]. A growing awareness on the impact of these envenomations has led to several initiatives by the WHO and diverse stakeholders in order to develop effective strategies for the prevention and control of this disease. A global strategy was developed, under the coordination of the WHO, aimed at reducing the impact of snakebite envenomations [4, 5]. One of the centerpieces of this strategy is the improvement of antivenom supply and access.

Antivenoms are usually produced by immunization of large animals, e.g. horses, donkeys or sheep with venom(s) of snakes inhabiting the country or region in which the antivenom is intended for use. After a few booster immunizations, the serum/plasma of the animals is obtained and fractionated to give either whole IgG or F(ab’)_2_ formulations [6]. The resulting preparation is then subjected to various quality control tests before being certified for use in the treatment of snakebite victims. The gold standard in the assessment of the preclinical efficacy of antivenoms is the neutralization of the lethal effect of venoms [6].

The antivenom potency assay requires, initially, the estimation of the Median Lethal Dose (LD_50_) of the venom(s) under study. This is followed by the neutralization of lethality assay, which is expressed as the Median Effective Dose (ED_50_) using *in vivo* murine assay, as recommended by the WHO [6]. In such tests, large number of mice are required. For example, 374 mice were needed per batch to assess venom LD_50_ and antivenom ED_50_ against five snake venoms [7], and 2,020 mice were used for testing the efficacy a pan-specific antiserum against 27 elapid venoms [8]. The routine testing of antivenom efficacy in quality control laboratories of manufacturers and regulatory agencies therefore demands a huge number of mice. Moreover, as new therapeutic alternatives are developed, such as new generation ‘synthetic’ antivenoms or chemical inhibitors, the need to validate these novel options requires the testing of their ability to neutralize the lethal effect of venoms [9–11].

There is a general ethical concern regarding this type of animal-based tests, since venoms inflict pain and distress to the animals. In various countries with a strong Buddhist tradition, and where snakebite envenomation is an important medical problem, doing experiments involving the killing of animals is largely prohibited. The 3Rs (Replacement, Reduction and Refinement) rationale in the use of experimental animals urges the development of *in vitro* tests that would substitute *in vivo* toxicity assays [12]. Various *in vitro* assays have been studied with the aim of reducing or replacing the *in vivo* murine assays. They include, among others, enzyme immunoassays [13–17], inhibition of *in vitro* coagulant effect [18], and inhibition of phospholipase A_2_ activity [19]. Moreover, the use of non-sensate fertilized chick embryo [20–22] and the use of *ex vivo* pharmacological models, such as isolated chick biventer cervicis [23] or isolated rat hemidiaphragm preparations [24, 25] have been also reported for assessing venom effects and neutralization by antivenoms. In the case of chick embryo, it is useful to assess toxicity of cytotoxic and hemotoxic venoms, but not of neurotoxic venoms since the six days embryo has not developed the target neuronal receptors.

The isolated nerve-muscle preparation has been very useful in the study of neurotoxic and myotoxic activities of venoms and purified toxins, and the ability of antivenoms to neutralize these effects [26–30]. However, few of these studies have correlated the results on nerve-muscle preparations with the *in vivo* lethality tests [29]. Despite their usefulness to study the action of neurotoxic venoms and toxins, and their neutralization by antivenoms from a research perspective, from a practical standpoint, these assays are technically difficult and time-consuming to set up in quality control laboratories for the routine assessment of antivenom efficacy.

More recently, an *in vitro* assay has been developed and is based on the high-affinity binding of snake postsynaptic α- neurotoxins to solubilized, purified nicotinic acetylcholine receptor (nAChR) [31, 32]. Since many elapid venoms exert their toxicity by binding to nAChR, hence causing neuromuscular blockade [33], this assay represents an *in vitro* correlate of the main mechanism of action of α-neurotoxin-rich venoms. The assay has been shown to give good correlation with *in vivo* estimation of LD_50_ of venoms and of ED_50_ of antivenoms when confronted with the venoms of the elapids *Naja kaouthia* (Thailand) [31], *Bungarus candidus* (Thailand) and *Naja naja* (Sri Lanka) [32].

As a follow up of these previous findings, we have expanded the analysis of correlation of this *in vitro* assay with the lethality of 20 neurotoxic elapid venoms, and also with the assessment of the neutralizing ability of a pan-specific elapid antiserum effective against 20 neurotoxic venoms belonging to 8 genera from 4 continents [34]. Our findings show that these *in vitro* assays give good correlation with both lethality and neutralization of lethality *in vivo* tests. This opens the possibility of using these assays in the assessment of antivenom efficacy in the case of neurotoxic venoms whose toxicity is predominantly based on post-synaptically acting α-neurotoxins.

## Materials and methods

### Chemicals and biochemicals

Chemicals and biochemical were obtained from Sigma Chemicals Co, St. Louis, Missouri, USA, unless otherwise indicated.

### Venoms, *T*. *californica* nAChR and horse pan-specific antiserum

Lyophilized venoms of *Naja siamensis* (Thailand), *Naja sputatrix* (Indonesia), *Naja philippinensis* (Philippines), *Naja atra* (Taiwan), *Naja melanoleuca* (Uganda), *Naja nigricollis* (Cameroon), *Naja haje* (Egypt), *Naja senegalensis* (Mali), *Dendroaspis angusticeps* (Tanzania), *Dendroaspis viridis* (Ghana) and *Dendroaspis polylepis* (Kenya) were purchased from Latoxan (Valence, France). The venoms of *Notechis scutatus* (Australia), *Pseudechis australis* (Australia), *Oxyuranus scutellatus* (Australia) and *Laticauda colubrina* (Bali, Indonesia) were obtained from Venom Supplies Pty Ltd. (Australia). *Bungarus multicinctus* venom (China) was from Yiwu City Jiashang Import & Export Co., Ltd., Zhejiang, China. *Bungarus candidus* (Indonesia) venom was from BioPharma, Bandung, Indonesia.

The venoms of Malayan Peninsula elapids including *Naja kaouthia* (Malaysia), *Naja sumatrana* (Seremban, Malaysia), *Ophiophagus hannah* (Seremban, Malaysia) and *Hydrophis schistosus* (Penang, Malaysia) were milked from adult snakes in the wild by Dr. Choo Hock Tan. Venoms of the wild caught specimens of *Naja oxiana* and *Naja naja* (both from Pakistan) were kind gifts from Dr. Naeem Quraishi. *Micrurus nigrocinctus* venom (Costa Rica) was provided by Prof. José María Gutiérrez. The venoms of *Naja kaouthia* (Thailand) pooled from several adult snakes of Thai origin were purchased from Queen Saovabha Memorial Institute (QSMI), The Thai Red Cross Society. The main *Naja kaouthia* postsynaptic neurotoxin 3 (NK3) was purified according to Karlsson et al [35].

*Torpedo californica* electroplaque was from Aquatic Research Consultant, California, USA. Nicotinic acetylcholine receptor (nAChR) from *T*. *californica* electroplaque was solubilized, extracted and purified as described by Lindstorm et al [36]. The anti-nAChR antisera were generated in rats as described by Ratanabanangkoon et al [31]. The pan-specific antiserum was prepared from horses immunized with 12 elapid toxin fractions/venoms as previously described [8].

### Estimation of the Median Lethal Dose (LD_50_) of neurotoxic venoms

The median lethal dose (LD_50_) of each of the 24 neurotoxic venoms was determined by intravenous (*i*.*v*.) injection in ICR mice (20–30 g, n = 4 per dose). The survival ratio was recorded after 24 h and LD_50_ was calculated using Probit analysis method, with variation depicted by the 95% confidence limits [37].

### *In vivo* neutralizing activity of horse pan-specific antiserum against various neurotoxic venoms

Neutralization of venom lethality by the pan-specific antiserum in mice was carried out as described previously [8]. Briefly, each venom was prepared in a volume of 50 μl 0.15 M NaCl (saline solution) to give a challenge dose of the venom corresponding to 5 x LD_50_ (or 2.5 x LD_50_ or 1.5 x LD_50_ depending on the venom). In the absence of the antiserum, these doses killed all the injected mice. The venom solution was then incubated with varying dilutions of the pan-specific antiserum using saline as diluent, to give a total volume of 250 μl. After incubation at 37°C for 30 minutes, the venom-antiserum mixtures were injected into the caudal vein of mice (20–30g, n = 4–5). The number of dead/alive mice was recorded after 24 h and ED_50_ was calculated using Probit analysis method, with variation depicted by the 95% confidence limits. The Median Effective Dose (ED_50_) of the antiserum against the venom was determined as the volume of antiserum (μl) that protected 50% of the challenged mice tested.

### *In vitro* nAChR binding assay

The basic assay conditions for the binding of purified nAChR to α-neurotoxin NK3 immobilized in microtiter plates (Polystyrene High Binding 3590, Costar), together with the optimal concentrations of reagents, was performed as described previously [31]. This assay involves first the addition of purified, solubilized nAChR to microtiter plates coated with neurotoxin NK3. Under the concentration and conditions used, the nAChR could bind maximally i.e., 100% to the NK3 coated plate. This nAChR binding is inhibited by elapid venoms containing α-neurotoxins. The *in vitro* Median Inhibitory Concentrations (IC_50_) of a venom is the concentration at which the nAChR binding was inhibited by 50%. Various concentrations of each venom were incubated with a predetermined optimal concentration of purified nAChR (0.930 μg/ml) at 25°C for 60 minutes. The mixture was then transferred to NK3 coated plates. Any unbound nAChR was washed off with 0.05% Tween 20 in phosphate buffer saline pH 7.2 and the amount of bound nAChR was determined by adding rat-anti-nAChR serum (1: 1,600), goat anti-rat IgG-HRP conjugate (Abcam) (1: 4,000) and TMB/H_2_O_2_ enzyme substrate (BioFX Laboratories, MN, USA). Absorbances at 450 nm were then recorded with microplate spectrophotometer (Multiskan Go, Thermo Scientific). The result was converted to percent binding of the nAChR to the plate [31, 32]. The concentration of the venom that reduced the nAChR binding by 50% corresponded to the IC_50_. The IC_50_ values were reported as mean ± standard deviation (n = 3).

### Neutralization of venoms by antiserum using the nAChR binding assay

In the *in vitro* assay of antiserum neutralization of neurotoxins, a fixed amount of each venom (corresponding to 5 x IC_50_ or 2.5 x IC_50_ or 1.5 x IC_50_) which could completely inhibit the binding of nAChR to the NK3 coated plate is used. The venom was pre-incubated with various volumes (0.078 μl–2.5 μl) of the pan-specific antiserum, and saline solution was added to maintain a constant final volume. Mixtures were incubated at 25°C for 90 min in a total volume of 480 μl. After this incubation, the antibodies, both free and bound to the venom toxins, were removed by ultrafiltration through 100 kDa MWCO ultrafiltration membranes (Amicon). The filtrates (126 μl), containing free α-neurotoxins, were then allowed to react with an optimal amount of nAChR (14 μl) at 25°C for 1 hr. The reaction mixtures were then added to NK3 coated microtiter plates. The amount of bound nAChR was then determined as described above for IC_50_ determination. The dose-response curves of horse serum volumes versus percent of nAChR binding were constructed. The *in vitro* neutralizing activities (ED_50s_) corresponded to the volumes of horse antiserum at which the nAChR binding was inhibited by 50% compared to wells incubated with non-immune horse serum in place of antiserum. The results were reported as mean ± standard deviation (n = 3).

### Miscellaneous procedures

The method described by Lowry et al [38] and the Bicinchoninic acid (BCA) Protein assay Kit (Pierce) were used to determine protein concentration, using bovine serum albumin as standard. GraphPad Prism 5.0 program was used in the calculation of *in vitro* IC_50_ and ED_50_ values and in generating the curves. Correlation analysis was made by linear regression with GraphPad Prism 5.0 software. The correlation coefficient was determined from the linear regression model; R^2^ is the square of the correlation coefficient. An R^2^ of 0.8–1.0 indicates a strong correlation between the two variables. The statistical significance of the correlation test was set at *p <0*.*05*.

### Ethics approval

The animal experiments in mice were carried out according to the guidelines of the Council for International Organizations of Medical Sciences (CIOMS) and were approved by the Institutional Animal Care and Use Committee (IACUC) of the University of Malaya (Ethical clearance No. 2016-190607/PHAR/R/TCH).

## Results

### The contents of α-neurotoxins in the elapid snake venoms studied

The 24 venoms analyzed in this study (Tables 1, 2 and 3) were from neurotoxic snakes of the family Elapidae belonging to 10 genera from 4 continents. Almost all of them are WHO category 1 most medically important snakes in their native countries or regions. Only two snakes, i.e., *Ophiophagus hannah* and *Micrurus nigrocinctus* are in WHO category 2 of less medically important snakes, although they have caused fatalities in humans. *Hydrophis schistosus* is a sea snake, and *Laticauda colubrina* is a sea krait. Twenty of these neurotoxic venoms have been shown by proteomics, biochemical and/or pharmacological studies to contain α-neurotoxins, being largely devoid of β-neurotoxins (Table 1), whereas others are known to contain α-neurotoxins, β-neurotoxins and other lethal toxins (Table 2) [39–42]. Four venoms (*Naja nigricollis*, *Oxyuranus scutellatus*, *Dendroaspis angusticeps* and *Pseudechis australis*) contain very low or no α-neurotoxins (Table 3).

**Table 1:** *In vivo* toxicity of *Naja* spp. and *Ophiophagus hannah* venoms and neutralization by the pan-specific antiserum.

#	Elapid species	Country of origin (WHO category)	Toxicity		Antiserum neutralization		Primary lethal toxins of the venoms ^e^	Ref. ^f^
			*In vitro* IC_50_ (μg/ml) ^a^	*In vivo* LD_50_ (μg/g) ^b^	*In vitro* ED_50_ (μl) ^c^	*In vivo* ED_50_ (μl) ^d^		
1	*Naja atra* (Taiwan cobra)	Taiwan (1)	0.3923 ± 0.0362	0.56* (0.37–0.84)	0.6507 ± 0.0679	50* (40.34–61.97)	**3FTx** SNTX and LNTX– 23.5% CTX– 52.9%	[43]
2	*Naja haje* (Egyptian cobra)	Egypt (1)	0.0653 ± 0.0031	0.09* (0.05–0.14)	0.9193 ± 0.1423	100* (80.68–123.94)	**3FTx** SNTX, LNTX and CTX detected (quantitative data not available)	[44]
3	*Naja kaouthia* (Monocled cobra)	Malaysia (1)	0.6936 ± 0.0960	0.90* (0.59–1.37)	1.1141 ± 0.0204	111.25* (73.28–168.89)	**3FTx** SNTX– 4.2% LNTX– 3.9% CTX– 45.7%	[45]
4	*Naja melanoleuca* (Forest cobra)	North Cameron (1)	0.2837 ± 0.0042	0.33* (0.22–0.51)	1.3186 ± 0.0021	141.36* (108.22–184.63)	**3FTx** SNTX– 7.3% LNTX– 13.4% CTX– 25.2%	[46]
5	*Naja naja* (Spectacled cobra)	Pakistan (1)	0.2818 ±0.0154	0.30* (0.27–0.33)	1.6489 ± 0.0468	175* (167.55–182.78)	**3FTx** SNTX– 4.7% LNTX– 21.6% CTX– 46.9%	[47]
6	*Naja oxiana* (Caspian cobra)	Pakistan (1)	0.7243 ± 0.0024	0.90* (0.59–1.37)	0.6667 ± 0.0214	60.43* (52.39–69.70)	N.A.	N.A.
7	*Naja philippinensis* (Philippine cobra)	Philippines (1)	0.0956 ± 0.0061	0.18* (0.12–0.27)	0.8358 ± 0.0054	100* (80.68–123.94)	**3FTx** SNTX– 44.6% CTX– 21.3%	[48]
8	*Naja senegalensis* (Senegalese cobra)	Mali (1)	0.2254 ± 0.0107	0.39 (0.25–0.61)	0.7159 ± 0.0365	78.95 (63.80–97.69)	N.A.	N.A.
9	*Naja siamensis* (Indochinese spitting cobra)	Thailand (1)	0.2319 ± 0.0190	0.28* (0.18–0.42)	1.8410 ± 0.0328	178.47* (161.28–197.49)	**3FTx** SNTX– 4.7% LNTX– 22.6% CTX– 33.8%	[49]
10	*Naja sputatrix* (Javan spitting cobra)	Indonesia (1)	0.6035 ± 0.0144	0.90* (0.59–1.36)	1.2376 ± 0.0028	125* (117.72–132.73)	**3FTx** SNTX– 7.9% LNTX– 0.5% CTX– 48.1%	[50]
11	*Naja sumatrana* (Equatorial spitting cobra)	Malaysia (1)	0.5071 ± 0.1540	0.5* (0.40–0.62)	1.0472 ± 0.0103	100* (80.68–123.94)	**3FTx** SNTX– 3.5% LNTX– 12.1% CTX– 44.2%	[51]
12	*Ophiophagus hannah* (King cobra)	Malaysia (2)	0.5541 ± 0.0129	0.90* (0.59–1.36)	1.0662 ± 0.0156	111.25* (73.28–168.89)	**3FTx** SNTX– 7.5% LNTX– 26.7% CTX– 0.5%	[52]

Note: SNTX and LNTX are α-neurotoxins.

Abbreviations: IC_50_, median inhibition concentration; LD_50_, median lethal dose; ED_50_, median effective dose; 3FTx, three-finger toxin; SNTX, short-neurotoxin; LNTX, long-neurotoxin; CTX, cytotoxin.

a Concentration of the venom that reduced the nAChR binding by 50%.

b Venom dose (μg/g) at which 50% of mice died.

c Volume (μl) of horse antiserum at which the nAChR binding was inhibited by 50 percent compared to wells incubated with non-immune horse serum in place of antiserum.

d Antiserum dose (μl) at which 50% of mice survived.

e Percentages indicated quantitative relative toxin abundances by total venom proteins.

f References to the principal toxins and their quantitative relative abundances.

*The data retrieved from Ratanabanangkoon et al., 2016 [8]

**Table 2:** *In vivo* toxicity of non-cobra/king cobra venoms and neutralization by the pan-specific antiserum.

#	Elapid species	Country of origin (WHO category)	Toxicity		Antiserum neutralization		Neurotoxins of the venoms ^e^	Ref. ^f^
			*In vitro* IC_50_ (μg/ml) ^a^	*In vivo* LD_50_ (μg/g) ^b^	*In vitro* ED_50_ (μl) ^c^	*In vivo* ED_50_ (μl) ^d^		
1	*Bungarus candidus* (Malayan krait)	Indonesia (1)	0.1329 ± 0.0170	0.11* (0.07–0.17)	1.0526 ± 0.0239	37.5* (34.27–41.04)	**3FTx** α-BTX and κ-BTX detected (quantitative data not available) **PLA_2_** β-BTX detected but (quantitative data not available)	[53]
2	*Bungarus multicinctus* (Many-banded krait)	China (1)	0.1214 ± 0.0074	0.014* (0.010–0.021)	0.9569 ± 0.0244	10.04* (9.55–10.55)	**3FTx** α-BTX– 6% κ-BTX– 2.4% **PLA_2_** β-BTX– 58.4%	[54]
3	*Dendroaspis polylepis* (Black mamba)	Kenya, South Africa (1)	0.1925 ± 0.0093	0.28 (0.16–0.51)	1.2913 ± 0.0127	152.63 (136.44–170.74)	**3FTx** SNTX– 3.7% LNTX– 13.2% Mambalgin– 1.4% Aminergic toxin—<0.1% L-type calcium blocker– 2.9% Dendrotoxin 61.1%	[42]
4	*Dendroaspis viridis* (Western green mamba)	Ghana, West Africa (1)	0.3032 ± 0.0130	0.15 (0.13–0.17)	1.0865 ± 0.0311	139.56 (104.98–185.53)	**3FTx** SNTX– 13.1% LNTX– 0.9% Mambalgin–<0.1% Aminergic toxin– 0.5% Mambin– 2.1% L-type calcium blocker– 7.7% Dendrotoxins– 5.6%	[55]
5	*Hydrophis schistosus* (Beaked sea snake)	Penang, Malaysia (Not classified)	0.0696 ± 0.0005	0.07 (0.05–0.09)	1.8114 ± 0.0967	201.49 (193.30–210.03)	**3FTx** SNTX– 55.8% LNTX– 14.7% **PLA_2_** Basic PLA_2_−21.4%	[41]
6	*Laticauda colubrina* (Yellow-lipped sea krait)	Bali, Indonesia (Not classified)	0.0782 ± 0.0034	0.15 (0.14–0.17)	1.3372 ± 0.0186	170.16 (153.72–188.37)	**3FTx** SNTX– 48.9% LNTX– 16.9% Cytotoxin– 0.3% **PLA_2_** Basic PLA_2_−33.2%	[40]
7	*Micrurus nigrocinctus* (Central American coral snake)	Costa Rica (2)	0.5876 ± 0.0624	0.51 (0.45–0.58)	1.2821 ± 0.1849	139.56 (104.98–185.53)	**3FTx** SNTX– 14.5% LNTX– 7.3% κ-BTX– 4.7% **PLA_2_** Acidic PLA_2_−5.2% Neutral PLA_2_−10.0%	[39]
8	*Notechis scutatus* (Australian tiger snake)	Southern Australia (1)	0.2235 ± 0.0203	0.09 (0.06–0.14)	1.4033 ± 0.0083	146.90 (129.05–167.21)	**3FTx** SNTX– 1.7% LNTX)– 4.0% **PLA_2_** Acidic—37.3% Basic—32.4% Neutral—4.8%	[56]

Note: SNTX and LNTX are α-neurotoxins.

Abbreviations: IC_50_, median inhibition concentration; LD_50_, median lethal dose; ED_50_, median effective dose; **3FTx**, three-finger toxin; SNTX, short-neurotoxin; LNTX, long-neurotoxin; CTX, cytotoxin; α-BTX, alpha-bungarotoxin; κ-BTX, kappa-bungarotoxin; β-BTX, beta-bungarotoxin; PLA_2_, phospholipase A_2_.

^a^ Concentration of the venom that reduced the nAChR binding by 50%.

^b^ Venom dose (μg/g) at which 50% of mice died.

^c^ Volume (μl) of horse antiserum at which the nAChR binding was inhibited by 50 percent compared to wells incubated with non-immune horse serum in place of antiserum.

^d^ Antiserum dose (μl) at which 50% of mice survived.

^e^ Percentages indicated quantitative relative toxin abundances by total venom proteins.

^f^ References to the principal toxins and their quantitative relative abundances.

*The data retrieved from Ratanabanangkoon et al., 2016 [8]

**Table 3:** *In vivo* toxicity of venoms containing very low amounts or devoid of α-neurotoxins (no binding to the nAChR in *the in vitro* assay).

#	Elapid species	Country of origin (WHO category)	Toxicity		Antiserum neutralization		Primary lethal toxins and other toxins of the venoms ^e^	Ref. ^f^
			*In vitro* IC_50_ (μg/ml) ^a^	*In vivo* LD_50_ (μg/g) ^b^	*In vitro* ED_50_ (μl) ^c^	*In vivo* ED_50_ (μl) ^d^		
1	*Dendroaspis angusticeps* (Green mamba)	Tanzania, East Africa (1)	-	1.53 (1.36–1.71)	-	>200 (not effective)	**3FTx** Mambalgin– 3.0% Aminergic toxin– 12.6% Mambin– 6.2% L-type calcium blocker–<0.1% **KSPI**– 16.3% Dendrotoxins– 8.4% **SVMP**– 6.7%	[57]
2	*Naja nigricollis* (Black*-*necked spitting cobra)	Cameroon (1)	-	0.75 (0.69–0.82)	-	156.57 (127.95–191.59)	**3FTx** SNTX– 0.4% CTX– 72.8% **PLA_2_**−21.9%	[58]
3	*Oxyuranus scutellatus* (Coastal Taipan)	Australia (1)	-	0.03 (0.02–0.04)	-	69.78 (52.49–92.76)	**3FTx** SNTX– 1.5% **PLA_2_** Acidic PLA_2_−45.7% Basic PLA_2_−33.7% **KSPI**– 7.8% **SVMP**– 5.2%	[59]
4	*Pseudechis australis* (King brown snake)	Australia (1)	-	0.31 (0.24–0.40)	-	76.32 (68.22–85.37)	**PLA_2_**−18.5% **KSPI**– 1.0% **SVMP**– 53.0%	[60]

Note: SNTX and LNTX are alpha-neurotoxins.

Abbreviations: IC_50_, median inhibition concentration; LD_50_, median lethal dose; ED_50_, median effective dose; **3FTx**, three-finger toxin; SNTX, short-neurotoxin; KSPI, Kunitz-type serine protease inhibitor; SVMP, snake venom metalloproteinase; PLA_2_, phospholipase A_2_.

^a^ Concentration of the venom that reduced the nAChR binding by 50%.

^b^ Venom dose (μg/g) at which 50% of mice died.

^c^ Volume (μl) of horse antiserum at which the nAChR binding was inhibited by 50 percent compared to wells incubated with non-immune horse serum in place of antiserum.

^d^ Antiserum dose (μl) at which 50% of mice survived.

^e^ Percentages indicated quantitative relative toxin abundances by total venom proteins.

^f^ References to the principal toxins and their quantitative relative abundances.

### Lethality assay and the inhibition of nAChR binding by α-neurotoxins of various elapid venoms

**Fig 1:**
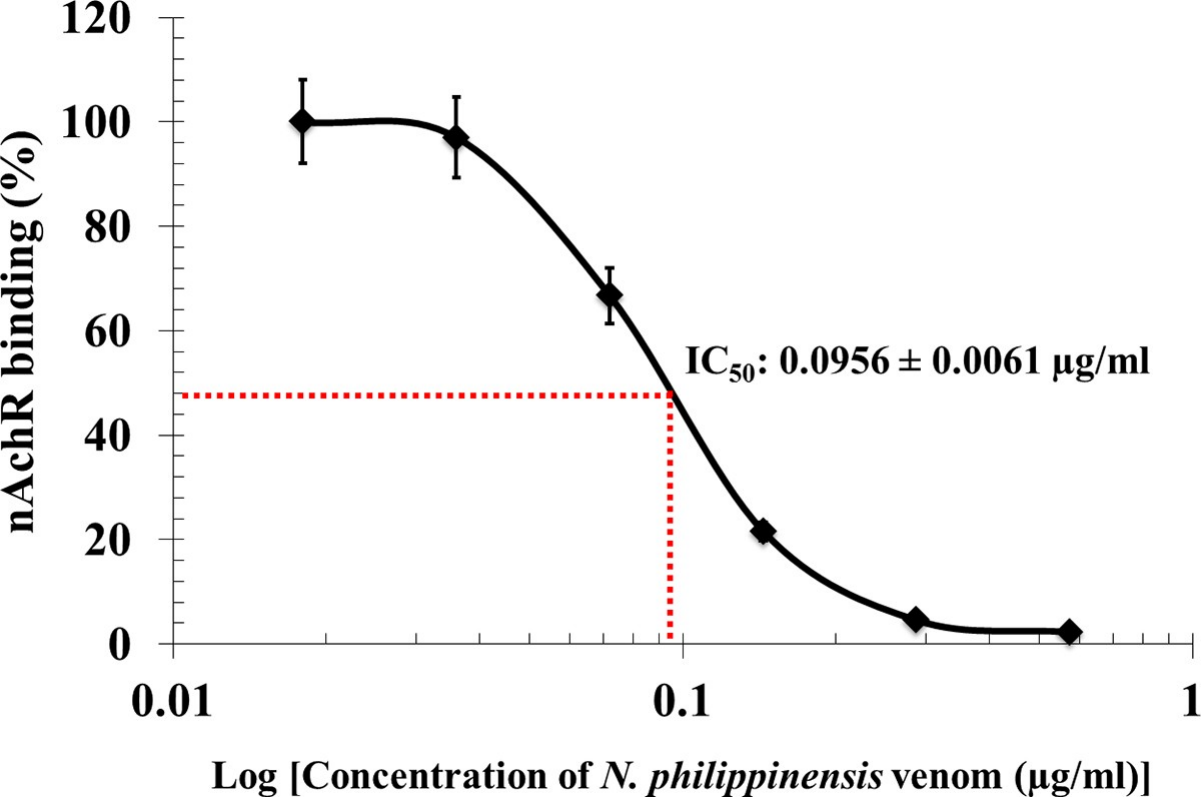
Determination of *in vitro* Median Inhibitory Concentration (IC_50_) of *N*. *philippinensis* venom in the assay of nAChR binding. Purified nAChR was incubated with various concentrations of *N*. *philippinensis* venom, and the mixture was added to plates coated with the neurotoxin NK3. The plate-bound nAChR was then detected with rat anti-nAChR antibody, followed by the addition of anti-rat IgG-HRP conjugate (see Materials and Methods for details). Results are presented as mean ± S.D. (n = 3).

Fig 1 shows the results of the inhibition of nAChR binding by α-neurotoxins in *Naja philippinensis* venom. When the concentration of the venom increases, more nAChR becomes occupied by the α-neurotoxins of the venom, and the binding of nAChR to the NK3 coated plate decreases. The 50% inhibition of nAChR binding (IC_50_) of *N*. *philippinensis* venom, as determined by regression, was 0.095 μg/ml.

**Fig 2:**
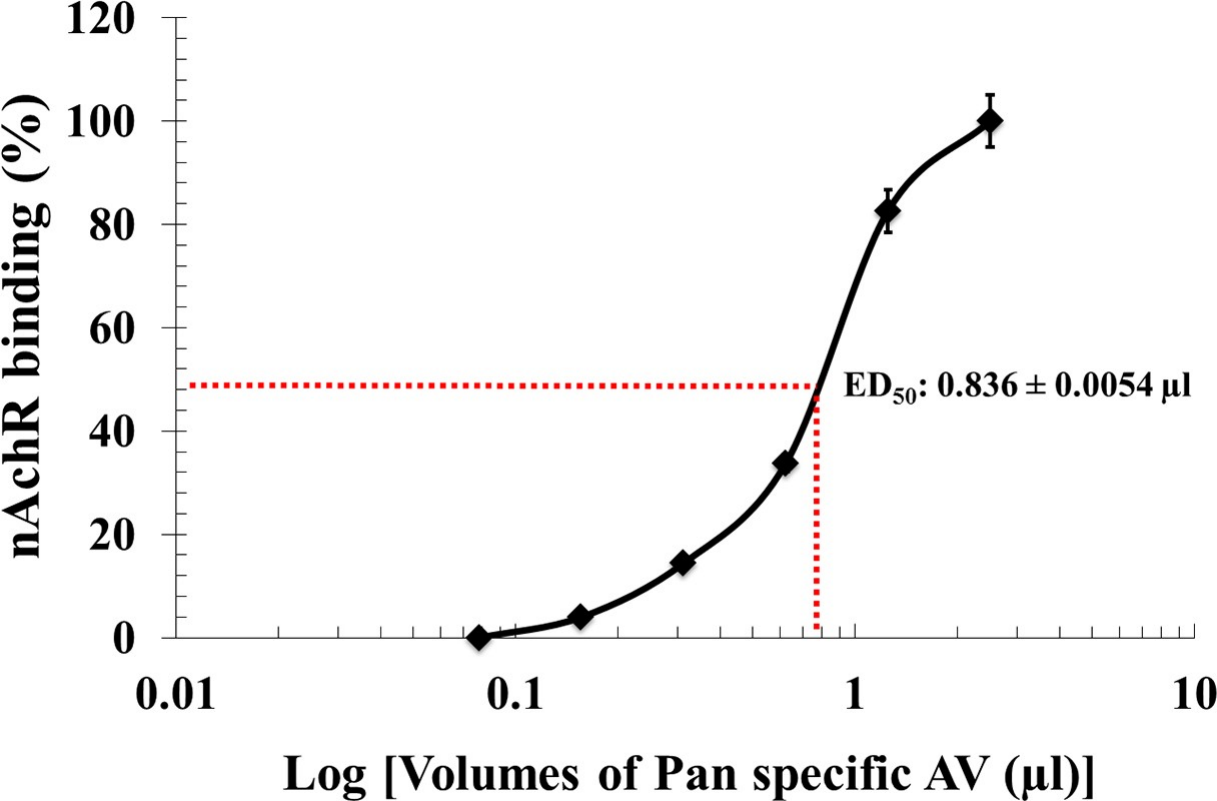
Determination of *in vitro* Median Effective Dose (*in vitro* ED_50_) of the pan-specific antiserum against *N*. *philippinensis* venom. Venom was incubated with various dilutions of pan-specific antiserum. After an ultrafiltration step, the filtrate was incubated with nAChR, and then added to the plates coated with NK3 neurotoxin. The plate-bound nAChR was detected by addition of rat anti-nAChR antibody (see Materials and Methods for details). Results are presented as mean ± S.D. (n = 3).

For the antiserum neutralization of the *in vitro* inhibition of nAChR binding, a dose of *N*. *philippinensis* venom corresponding to 2.5 x IC_50_, i.e. 0.237 μg/ml, was used. The venom solutions were incubated with varying volumes of antiserum (0.078 to 2.5 μl), as described in the methods and the binding of nAChR to the NK3 coated plate was then assessed. The volume of the antiserum that resulted in 50% inhibition of nAChR binding is the *in vitro* ED_50_ of the antiserum, in this case corresponding to 0.836 μl (Fig 2).

Following these methodologies, the results of the *in vivo* LD_50s_ and the *in vitro* IC_50s_ of the 20 neurotoxic venoms are shown in Tables 1 (no.1-12) and 2 (no.13-20). The most lethal venom was *Hydrophis schistosus* (LD_50_ of 0.07 μg/g), whereas the least potent venoms were *Naja sputatrix* (Indonesia) and *Naja kaouthia* (Malaysia) with LD_50_ of 0.90 μg/g. *Bungarus multicinctus* from China also possessed potent venom with LD_50_ at 0.014 μg/g, while that of *Bungarus candidus* from Indonesia was 0.11 μg/g.

The *in vitro* inhibition of the nAChR binding to NK3 coated on the microtiter plate, expressed as IC_50_ values of various elapid venoms are shown in Tables 1 and 2. The *Naja haje* (Egypt) venom showed the highest activity (IC_50_ = 0.0653μg/ml), whereas the least active venom was *Naja oxiana* (Pakistan) (IC_50_ = 0.7243 μg/ml).

**Fig 3:**
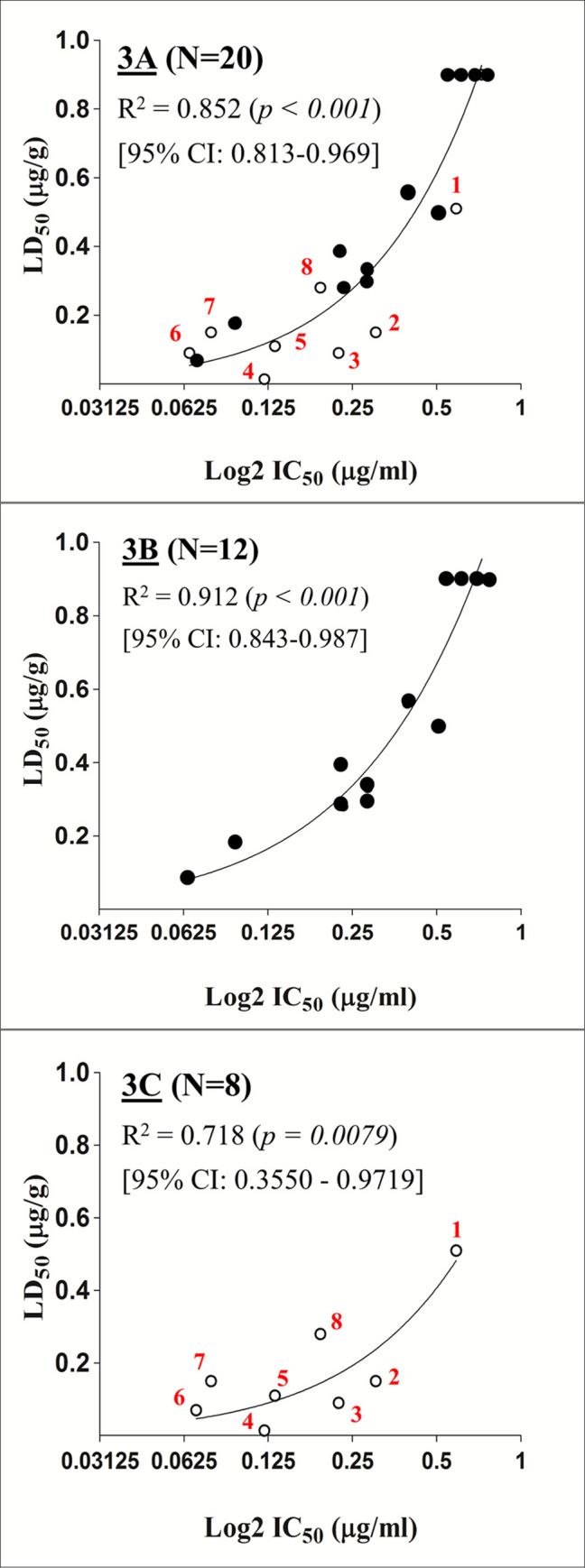
The correlation plots between the *in vivo* lethality (LD_50_) and the *in vitro* nAChR binding inhibition (IC_50_) of various neurotoxic venoms. (3A) all 20 neurotoxic venoms; (3B) 12 *‘Naja spp*.’ venoms; (3C) 8 ‘non-*Naja spp*.’ venoms. The ‘*Naja spp*.’ venoms are denoted by (●) while the ‘non-*Naja spp*.’ venoms are denoted by (○). The identities of the ‘non-*Naja spp*.’ snakes are: #1, *M*. *nigrocinctus*; #2, *D*. *viridis*; #3, *N*. *scutatus*; #4, *B*. *multicinctus*; #5, *B*. *candidus*; #6, *H*. *schistosus*; #7, *L*. *colubrina*; #8, *D*. *polylepis*.

The correlation between the *in vivo* LD_50s_ and the *in vitro* IC_50s_ for the 20 venoms studied is shown in Fig 3A. The correlation R^2^ is 0.8526 (*p* < 0.001) which is statistically significant. The 20 neurotoxic venoms tested can be divided into two groups, according to their relative content of α-neurotoxins: (a) One group that contains mainly α-neurotoxins without pre-synaptically active β-neurotoxins (the *‘Naja spp*.’ Group). This group consists of 11 venoms from *Naja* species. In addition, we included the venom of *O*. *hannah* within this group owing to its similar pharmacological and toxin profiles as that of other venoms in this group (Table 1). (b) The other group includes venoms that contain highly lethal pre-synaptic β-neurotoxins in addition to α-neurotoxins (the ‘non-*Naja spp’* group) (Table 2). The correlation between the *in vivo* LD_50s_ and the *in vitro* IC_50s_ of the *‘Naja spp*.’ venoms gives a correlation R^2^ of 0.912 (*p* <0.001) which is statistically significant (Fig 3B). The corresponding correlation of the ‘non-*Naja spp*.*’* group is slightly lower at a R^2^ value of 0.718 (*p* < 0.0079), which is also statistically significant (Fig 3C).

### The *in vivo* and *in vitro* assays of neutralization by pan-specific antiserum against the 20 neurotoxic venoms

**Fig 4:**
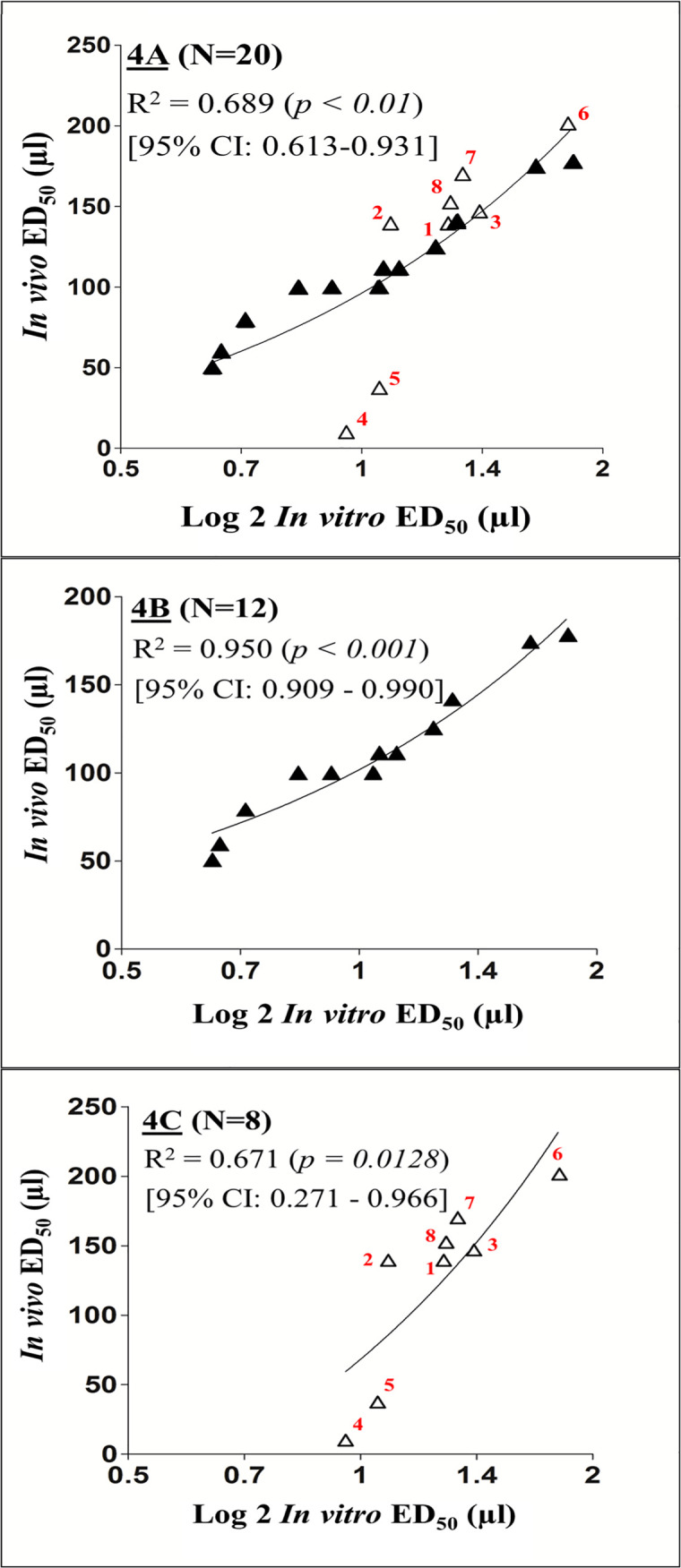
The correlation plots between the *in vivo* lethality neutralization potency (ED_50_) and the *in vitro* inhibition of nAChR binding (ED_50_) by a pan-specific antiserum. (4A) all 20 neurotoxic venoms; (4B) 12 *‘Naja spp*.’ venoms; (4C) 8 ‘non-*Naja spp*.’ venoms. The ‘*Naja spp*.’ venoms are denoted by black triangles while the ‘non-*Naja spp*.’ venoms are denoted by white triangles. The identities of the ‘non-*Naja spp*.’ venoms are: #1, *M*. *nigrocinctus*; #2, *D*. *viridis*; #3, *N*. *scutatus*; #4, *B*. *multicinctus*; #5, *B*. *candidus*; #6, *H*. *schistosus*; #7, *L*. *colubrina*; #8, *D*. *polylepis*.

Tables 1 and 2 show the *in vivo* ED_50s,_ i.e. the neutralization of lethal effect, of the horse pan-specific antiserum against the 20 neurotoxic venoms. The antiserum was shown to neutralize the lethality of all the 20 venoms with different degrees of effectiveness. The *in vitro* potency assays of the pan-specific antiserum against these 20 venoms *(in vitro* ED_50s_) are shown in Tables 1 and 2. The correlation plot between the *in vivo* ED_50s_ and the *in vitro* ED_50s_ of the 20 venoms, shown in Fig 4A, gives the R^2^ of 0.689 (*p* < 0.01) which was statistically significant. When the 20 neurotoxic venoms were divided into the *‘Naja spp*.’ and ‘non-*Naja spp*’ groups, the correlation between the *in vivo* and *in vitro* antiserum potency against the *‘Naja spp*.’ group was 0.950 (*p* < 0.001) which is statistically highly significant (Fig 4B). The corresponding correlation for the ‘non-*Naja spp*.’ group which consists of 6 genera is lower at 0.671 (*p* < 0.0128) but still remains statistically significant (Fig 4C). Two venoms, those of *B*. *multicintus* (#4) and *B*. *candidus* (#5) seemed to deviate from the correlation line of other venoms in the plot (Fig 4A and 4C).

### *In vitro* nAChR binding of neurotoxic venoms containing low or no α-neurotoxins

**Fig 5:**
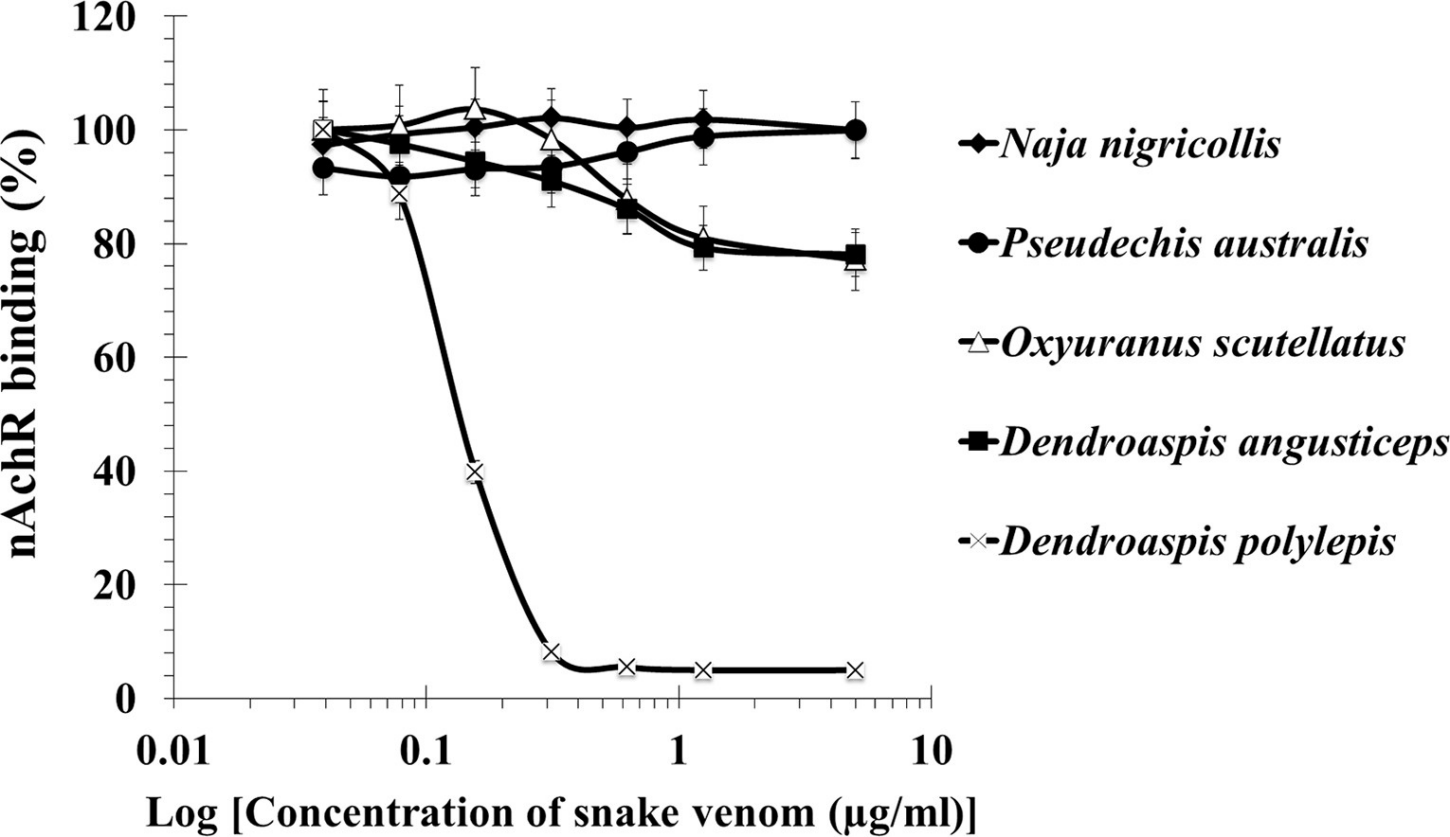
Inhibition of nAChR binding to NK3 coated microtiter plate by various neurotoxic venoms with very low or no α-neurotoxins. *D*. *polylepis* venom is used as a positive control since it contains α-neurotoxins. Results are presented as mean ± S.D. (n = 3).

There are 4 neurotoxic venoms (no. 21–24 in Table 3) that failed to inhibit the binding of nAChR to the NK3 coated plate in the *in vitro* IC_50_ assay. These venoms were *N*. *nigricollis* (Cameroon), *P*. *australis* (Australia), *O*. *scutellatus* (Australia) and *D*. *angusticeps* (Tanzania) (Fig 5). Although the venoms of *O*. *scutellatus* and *D*. *angusticeps* showed a partial inhibition of about 20% of the nAChR binding, the *in vitro* IC_50s_ (and consequently, the *in vitro* ED_50s_ of the antiserum) of these four venoms could not be determined. These venoms have been shown by proteomics to contain no (i.e., *P*. *australis* and *D*. *angusticeps*) or low content of α-neurotoxins (*N*. *nigricollis* and *O*. *scutellatus*) [57–60] (Table 3). The venom of *O*. *scutellatus* or the coastal Taipan, contains approximately 1.5% of short-chain α-neurotoxins [57]. Fig 5 also shows the inhibition of *D*. *polylepis* venom, which contains α-neurotoxins [42, 56], and served as a positive control.

## Discussion

To reduce the use of mice required for the assessment of venom toxicity (lethality) and antivenom neutralizing ability, the WHO urges the development of alternative *in vitro* tests that could substitute animal-based assays [6]. We report here an *in vitro* assay based on the α-neurotoxin-nAChR binding. The post-synaptically acting α-neurotoxins, which bind with high affinity to the nAChR, are abundant in many elapid venoms and play a key role in neurotoxic snakebite envenomation [33, 61]. We showed that this assay is highly useful to assess the ability of neurotoxic venoms in binding to solubilized, purified nAChR, and the capacity of antivenoms to inhibit this binding. By using a large number of venoms from a variety of elapid species, our results show that the two *in vitro* assays correlated well with the corresponding *in vivo* tests. Therefore, these *in vitro* assays could be adapted for research and quality control laboratories as convenient surrogate tests to assess the neurotoxic activity of post-synaptically acting venoms and its neutralization by antivenoms, hence reducing the large scale use of mice in these tests.

### *In vitro* nAChR binding of venom α-neurotoxins and *in vivo* venom toxicity

It was shown that the IC_50s_ values of the 20 elapid venoms for the *in vitro* nAChR binding assay correlate well with the murine LD_50s_ of these venoms. This correlation was higher in the ‘*Naja spp*.’ group than that in the ‘non-*Naja spp*.’ group. The ‘*Naja spp*.’ group, consisting of eleven *Naja spp*. and *O*. *hannah* venoms, contain α-neurotoxins and cytotoxins, but no β-neurotoxins. The cytotoxins, also known as cardiotoxins, are less toxic than α-neurotoxins, with LD_50_ values of 1.0–1.75μg/g in mice [45, 49, 62], and are usually involved in local tissue necrosis but not lethality in the victims. The high correlation observed with the *‘Naja spp*.’ group is due to the fact that the α-neurotoxins are responsible for both the *in vitro* nAChR binding and in the *in vivo* lethality. This is not the case for the ‘non-*Naja spp*.*’* group of venoms, which contain, in addition to α-neurotoxins, highly lethal β-neurotoxins (LD_50_ about 10 ng/g mice) [63–66] and other lethal toxins which also contribute to lethality in mice.

The scattering of the data points in the correlation plot of the ‘non-*Naja spp*.’ group is probably attributed to the heterogeneity of the variety of toxins present in the venoms of this group, e.g. α-, β-, κ- neurotoxins in the *B*. *candidus* venom; dendrotoxins and fasiculins in the venoms of *D*. *viridis* and *D*. *polylepis;* and presynaptically-acting neurotoxic PLA_2_s in several of these venoms, particularly in those of *Bungarus sp*. (Table 2). These non-α-neurotoxins play a role in the overall toxicity of the ‘non-*Naja spp*.’ venoms, hence explaining the lower correlation between LD_50_ and IC_50_ values. Nevertheless, the correlation is still significant, underscoring the role of α-neurotoxins in the lethal action of these venoms as well. In contrast, the nAChR binding assay is not useful in the case of venoms which lack, or have very low amount of α-neurotoxins (Table 3).

Regarding the interaction of α-neurotoxins and nAChR in the described *in vitro* assays, the following aspects deserve consideration:

- Some of these elapid venoms contain short and/or long α-neurotoxins which exhibit different nAChR binding kinetics and affinity [67, 68] and could possibly affect the IC_50_ determination. However, the experimental conditions of the assay using a 60 min pre-incubation time between the venom and nAChR, would allow for complete binding of α-neurotoxins, short or long, to the receptor.
- b. Since the nAChR-α-neurotoxins interaction is highly specific and of high affinity, the assay may be used to detect the presence or absence of ‘functional’ α-neurotoxins in the venoms in terms of their ability to bind to nAChR. This allows the distinction between three-finger toxins that bind to the receptor from those that do not bind. For example, proteomics analysis of *N*. *nigricollis* venom found short chain α-neurotoxins to be only 0.4% of the venom protein and 73% of three-finger toxins of the cytotoxin type [58]. In agreement, this venom did not show binding to nAChR in our experiments (Fig 5 and Table 3).
- The observation that the 20 neurotoxic elapid venoms inhibit the binding of nAChR to the NK3 coated plate indicates that α-neurotoxins of these venoms interact with high affinity to nAChR purified from *T*. *californica*. The fact that there is correlation between this *in vitro* assay using nAChR from ray electric organ, and lethality in mice, highlights the structural similarity between these receptors in these taxa, and the value of using ray receptor for assessing venom post-synaptic neurotoxicity. The immunological similarities between these receptors have been described [69].

### Inhibition of *in vitro* nAChR binding by antiserum and *in vivo* neutralization of venom toxicity

When assessing the ability of the pan-specific antiserum to neutralize the elapid venoms, a highly significant correlation between the *in vivo* and *in vitro* results was found, especially for the ‘*Naja spp*.’ group. These observations underscore the key role of α-neurotoxins in the overall toxicity of the venoms, and the fact that inhibition of the toxins binding to nAChR abrogates their *in vivo* lethal activity.

The correlation between the *in vitro* and *in vivo* neutralization assays of the ‘non*-Naja spp*.’ group was lower than that in the case of the ‘*Naja spp*.’ group venoms. This is probably due to the heterogeneity of the lethal toxins present in these venoms as previously discussed in the lethality assays above. Furthermore, the amount of specific antibodies present in the pan-specific antiserum against these lethal components is likely to vary depending on the cross reaction of the antibodies against the 6 heterologous venoms used in the assay, as discussed below.

Interestingly, the plots of *in vivo* ED_50_ vs. *in vitro* ED_50_ against the two *Bungarus* venoms, i.e. *B*. *candidus* (Indonesia) and *B*. *multicinctus* (China), deviated from the correlation line. When these two *Bungarus*, venoms were excluded from the analysis, the correlation between the assays was quite high (R^2^ = 0.9046, *p* < 0.001). *Bungarus spp*. venoms have a high concentration of the presynaptically-acting PLA_2_ heterodimeric β-bungarotoxin [53, 54]. Hence, despite the fact that these venoms also contain α-neurotoxins, β-bungarotoxins are likely to play a dominant role in lethality, although they do not bind to nAChR. This may explain the deviation of these venoms in the correlation curve. For the majority of venoms tested, *i*.*e*. those of the *‘Naja spp*.’ and ‘non-*Naja spp*.’ groups, the correlation observed in the neutralization of *in vitro* nAChR binding and the *in vivo* lethality support the contention that this *in vitro* assay could become a useful surrogate test to assess the neutralizing efficacy of antivenoms against these neurotoxic venoms. To the best of our knowledge, this nAChR binding assay is the first *in vitro* test to show a significant correlation with the *in vivo* mouse lethality test in the assessment of the neutralizing efficacy of antivenoms against elapid neurotoxic venoms. It would be relevant to expand these studies to other elapid venoms whose toxicity relies predominantly on the action of α-neurotoxins. In contrast, this assay is unsuitable in the case of venoms whose toxicity is based on toxins different from α-neurotoxins, such as those grouped in Table 3.

### Advantages of the nAChR binding assay

- By significantly reducing the use of mice, the *in vitro* assay used in this work fits within the 3Rs principles and, therefore, follows the trend proposed by the WHO for preclinical testing of antivenoms [6]. Furthermore, the assay should not encounter ethical and religious restrictions.
- The *in vitro* assays are simple and easy to perform; one researcher or technician can handle dozens of venom and antivenom samples without difficulty. Thus, being high-throughput tests, they allow the assessment of many venom and antivenom samples within a couple of days.
- The assays are much cheaper when compared to the *in vivo* mouse assays, owing to the high cost of mice and their maintenance.
- Being an *in vitro* test, where parameters can be readily controlled, it shows less variability than the *in vivo* mouse lethality assay.
- Although the final preclinical test of antivenom neutralizing efficacy will continue to be the mouse lethality neutralization assay, which is the gold standard of antivenom testing [6], the *in vitro* assay described may be used for other phases of antivenom production. These include in-process assessment of antivenom efficacy, and testing the samples of sera from immunized horses, in order to define whether a horse has achieved a satisfactory antibody response. These will greatly reduce the number of mice used in antivenom production laboratories. Likewise, this assay will allow the high throughput screening of novel inhibitors and recombinant antibodies against neurotoxic elapid venoms. In addition, the comparative analysis of nAChR binding by different venoms can help in the selection of venom doses to be used in *in vivo* lethality assays, again reducing the number of mice utilized. Taken together, these advantages will decrease animal suffering and will speed up the development of novel antivenoms and inhibitory substances.

### Possible drawbacks of the *in vitro* nAChR binding assay

A possible drawback of this procedure lies in the fact that the interaction of α-neurotoxins with nAChR has been shown to be prey-selective and hence varies depending on the taxon of origin of the receptor (amphibian, lizard, snake, bird or rodent) [70,71]. Likewise, a venom like *N*. *nigricollis*, which showed little affinity for ray nAChR in our study, binds to amphibian receptor [70]. Thus, the selection of the source of nAChR should be carefully considered. Nevertheless, the fact that we observed a high correlation between the nAChR binding assay and the *in vivo* mouse lethality assay strongly suggests that nAChR from ray electric organ is a suitable model for assessing post-synaptic neurotoxicity of snake venoms.

The major hurdle of these *in vitro* assays is the need to have solubilized, purified nAChR and the rat anti-nAChR antibodies which are currently not commercially available. However, these reagents can be prepared using standard biochemical techniques [36]. Once these reagents are prepared, they are quite stable and can be used for a large number of samples since each assay requires only nanogram amounts of the receptor and sub-microliter volume of antiserum. A promising alternative is the use of mimotopes and peptides derived from nAChR which bind to α-neurotoxins [70, 72]. The introduction of these synthetic peptides, if shown to bind specifically and with high affinity to α-neurotoxins, in this type of assay will avoid the need to obtain the receptor from rays or eels.

The present *in vitro* assay is based on the interaction of the venom α-neurotoxins and the nAChR. Thus, the assay does not work for venoms whose toxicity is not based on the action of α-neurotoxins. Proteomic and toxicity score analysis of venoms [73] for identifying the most active neurotoxins will allow the identification of venoms where α-neurotoxins do not play a key role, and for which the *in vivo* lethality assay has to be used.
